# Trophic feeding for very preterm or very low birth weight infants

**DOI:** 10.1186/1824-7288-41-S1-A3

**Published:** 2015-09-24

**Authors:** Elisa Civardi, Francesca Garofoli, Chryssoula Tzialla, Margherita Pozzi, Mauro Stronati

**Affiliations:** 1 Neonatal Intensive Care Unit, Fondazione IRCCS Policlinico San Matteo, Pavia, Italy; 2 NeonatalImmunologyLaboratory, Neonatal Intensive Care Unit, Fondazione IRCCS Policlinico San Matteo, Pavia, Italy

## 

Feeding very low birth weight (VLBW) or very preterm infants poses a unique challenge due to the immaturity of gastrointestinal tract. Early nutrition is crucial for improving optimal growth, long-term outcome and to decrease morbidities. The goal is to achieve a growth rate similar to fetal growth in utero.

Trophic feeding (TF) of preterm infants was introduced in the late 1980s in an attempt to overcome the lack of gastrointestinal stimulation during total parenteral nutrition. Alternative names include gut priming, minimal enteral nutrition and early hypocaloric feeding. TF is defined as providing nutritionally insignificant volumes of enteral substrate to compromised infants in order to stimulate and supply nutrients to the developing gastrointestinal system.

Minimal enteral nutrition stimulates gut hormones, promotes structural and functional intestinal maturation, decreases indirect hyperbilirubinemia and cholestatic jaundice. TF supports gastrointestinal disaccharidase activity, blood flow and microbial flora.

Clinical benefits include improved feeding tolerance, better weight gain, improved bone mineralization, reduced systemic sepsis and shorter hospital stay; moreover minimal enteral feeding facilitates a smooth and rapid transition from parenteral to enteral nutrition.

Nevertheless several studies showed beneficial effects, results could not be confirmed in a meta-analysis [1].

On the other hand, it is important to emphasize that metaanalysis did not suggest any harmful effects and no increased incidence of necrotizing enterocolitis, while lack of enteral nutrition causes gut atrophy and bacterial translocation [2].

Despite considerable research, there are several outstanding questions regarding TF: how the timing of introduction and the rate of progression of enteral feeding may affect clinical outcome? Which babies should be treated? What is the optimal duration and volume?

However, the following recommendations can be made on the basis of the published studies. Almost all the very low birthweight infants unable to tolerate substantial milk feeds, should be considered for TF. Exclusions are infants with necrotizing enterocolitis or congenital gastrointestinal abnormalities, such as gastroschisis. As delaying feeding appears to confer no advantage, it is reasonable to start TF on day 1 or 2 of life, providing the infant is stable. It appears that 0.5-1 ml/kg/h is a safe and effective volume. The optimal duration of TF is difficult to be recommended, and rather than specify a set time, regardless of clinical status, it is probably more sensible to suggest continuing TF until the infant can safely tolerate substantial volumes of milk. Breast milk, if available, should be preferred to formula [3].
